# *In vitro* characterisation of a pleconaril/pirodavir-like compound with potent activity against rhinoviruses

**DOI:** 10.1186/s12985-015-0330-4

**Published:** 2015-07-14

**Authors:** Céline Lacroix, Samuela Laconi, Fabrizio Angius, Antonio Coluccia, Romano Silvestri, Raffaello Pompei, Johan Neyts, Pieter Leyssen

**Affiliations:** Department of Microbiology and Immunology, Laboratory for Virology and Chemotherapy, KU Leuven, Rega Institute for Medical Research, B‐3000 Leuven, Belgium; Department of Biomedical Sciences, University of Cagliari, I-09124 Cagliari, Italy; Istituto Pasteur-Fondazione Cenci Bolognetti, Dipartimento di Chimica e Tecnologie del Farmaco, Sapienza Università di Roma, Piazzale Aldo Moro 5, I-00185 Rome, Italy

## Abstract

**Background:**

Rhinovirus infections do not only cause common colds, but may also trigger severe exacerbations of asthma and chronic obstructive pulmonary disease (COPD). Even though rhinoviruses have been the focus of extensive drug development efforts in the past, an anti-rhinoviral drug still has to make it to the market. In the past, the viral capsid protein VP1 has been shown to be an important target for the development of antiviral molecules. Furthermore, many different chemical scaffolds appear to possess the properties that are required to inhibit virus replication by this mechanism of action. I-6602, an analogue of the rhinovirus inhibitor pirodavir, was previously identified as a potent inhibitor of rhinovirus infection. Here, we describe the antiviral activity of its analogue ca603, a molecule with a modified linker structure, and corroborate its mechanism of action as a capsid binder.

**Findings:**

The molecule ca603 shows antiviral activity against a panel of rhino-and enteroviruses. Cross-resistance is observed against viruses with mutations that render them resistant to the inhibitory effect of the capsid binder pleconaril and thermostability assays demonstrate that the compound binds and stabilizes the viral capsid. Binding of the molecule to the VP1 protein is corroborated by *in silico* modeling.

**Conclusions:**

It is confirmed that ca603 inhibits rhinovirus replication by interaction with the VP1 protein and, by this, allows to further expand the chemical diversity of capsid-binding molecules.

**Electronic supplementary material:**

The online version of this article (doi:10.1186/s12985-015-0330-4) contains supplementary material, which is available to authorized users.

## Body of text

**Fig. 1:**
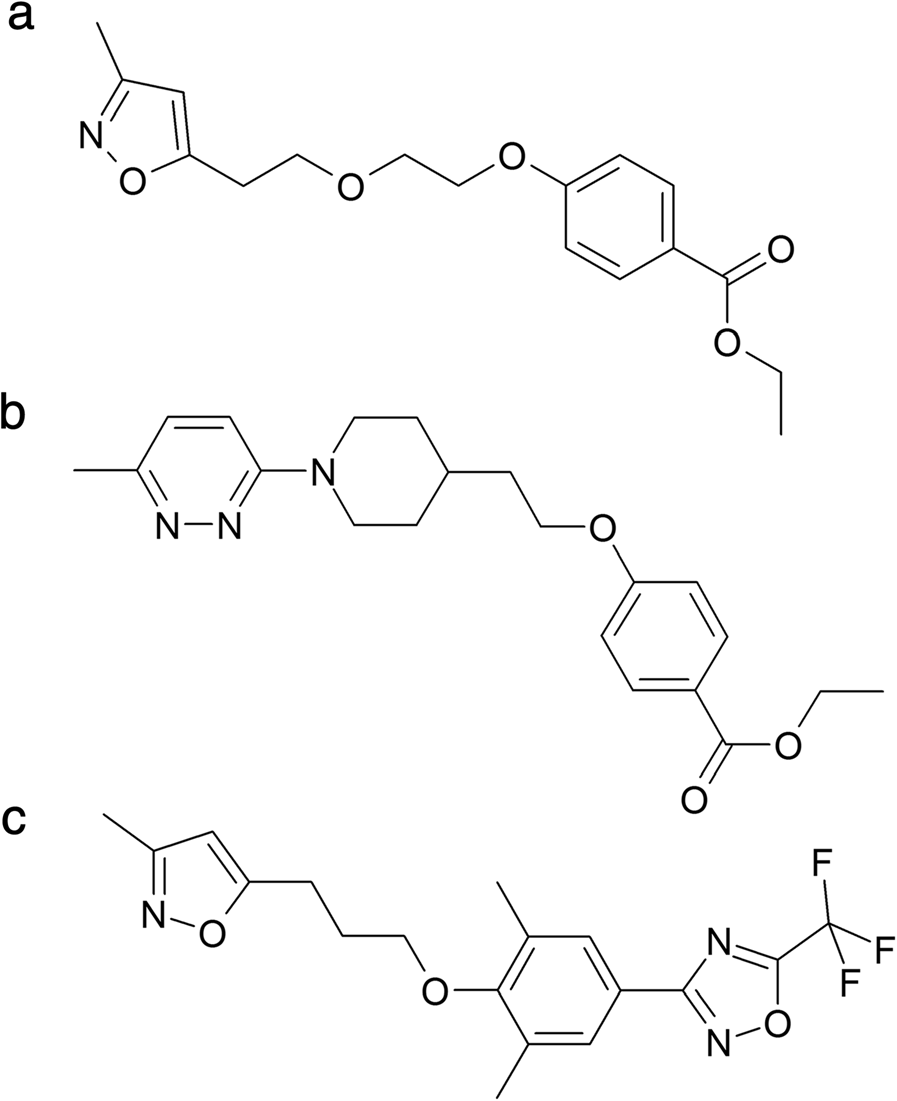
Structural formulae of (**a**) ca603, (**b**) pirodavir, and (**c**) pleconaril

The genus *Enterovirus* comprises several human pathogens with a substantial clinical impact on society like poliovirus (PV), rhinoviruses (HRV) and enterovirus 71 (EV71) [1]. Rhinoviruses cause common colds in healthy people but may trigger exacerbations in patients with asthma and COPD [2, 3]. Vaccination against rhinovirus infection is not yet feasible due to the large number of serotypes. Treatment with small-molecule inhibitors therefore seems to be the best possible option to lower the burden of this disease. To date, the rhinoviral capsid and protease are the best characterized antiviral targets. However, a drug has still to be approved for clinical use [1]. In the past, several early-stage rhinovirus inhibitors, referred to as capsid binders, were discovered. Pleconaril, pirodavir and vapendavir are the most extensively studied capsid binders (Fig. 1). In 2002, the New Drug Application for pleconaril (Schering-Plough) as drug against the common cold was rejected by the FDA, mainly because of safety reasons [4, 5]. Pirodavir (Janssen Pharmaceutica) inhibits *in vitro* the replication of both HRV-A,–B and, to a lesser extend, that of other enteroviruses [6]. Prophylactic treatment with pirodavir reduced the frequency of experimental rhinovirus infections and subsequent clinical colds only when the drug was administered intranasally six times a day [7]. Oral delivery of the ester-derivate pirodavir is not feasible given the fact that it rapidly hydrolyses to an inactive acid. No reduction of clinical symptoms was observed in the subsequent therapeutic trial with experimental rhinovirus infection. Although pirodavir treatment of naturally acquired rhinovirus infections significantly reduced virus shedding during the treatment period, no positive outcome on the clinical signs and respiratory symptoms was observed [8]. Furthermore, in all trials, pirodavir treatment was associated with an unpleasant taste and the more frequent presence of blood in the nasal mucus. Vapendavir (Biota Holdings), a benzisoxazole analogue of pirodavir, is currently in clinical development for the treatment of rhinovirus-induced asthma exacerbations [9]. In the past, we explored the antiviral activity of a panel of pirodavir analogues with modifications of the central hydrocarbon chain [10, 11]. Here, we report on the particular characteristics of the antiviral activity and mode of action of a novel and the until now most potent and least toxic analogue in this series (Fig. 1). Compound ca603 has been designed based on the structure of I-6602 and was synthesized at ASM Company (Burgwedel, Germany).

The antiviral activity of ca603 was assessed in a multi-cycle, virus-cell-based cytopathic effect (CPE) reduction assay in HeLa cells [12]. The compound proved to be active against HRV strains from both the HRV-A (HRV09, HRV29, HRV85 and HRV89) and HRV-B group (HRV14, HRV70 and HRV86) (Table 1) with a 50 % effective concentrations (EC_50_’s) ranging from 0.01 μM to 15 μM. Although ca603 exerted *in vitro* antiviral activity against EV71, PV1 and echovirus 11 (ECHO11), the compound was not able to induce complete protection against virus-induced CPE at non-toxic concentrations (Table 1).

**Table 1:** Activity of ca603 and pleconaril against representative viruses from HRV-A and B; and EV-A,-B and-C

Virus strain		EC_50_ (ca603) (μM)	EC_50_ (pleconaril) (μM)
HRV-A	HRV02	>313	0.2 ± 0.1*
	HRV09	8.9 ± 4.2*	0.2 ± 0.1*
	HRV29	1.7 ± 0.04*	0.1 ± 0.1*
	HRV63	>157	0.1 ± 0.1*
	HRV85	3.7 ± 2.0*	0.1 ± 0.1*
	HRV89	0.9 ± 0.8*	0.7 ± 0.3*
HRV-B	HRV14	0.10 ± 0.03*	0.3 ± 0.2*
	HRV42	8.2 ± 4.7	>26
	HRV70	15 ± 8*	4 ± 3.6
	HRV86	0.01 ± 0.003*	0.1 ± 0.1*
EV-A	EV71	74 ± 5	>52
EV-B	CVB3	>313	>131
EV-B	ECHO11	2.5 ± 1.6	1.7 ± 0.4
EV-C	PV1	25 ± 7	>131

Antiviral activity was determined in a CPE reduction assay with MTS read-out. EC_50_ = median 50 % effective concentration ± MAD from dose response curves set up from ≥ four experiments of which at least two independent. * = 100 % inhibition of virus-induced cytopathic effect can be achieved with this compound (as determined by microscopic inspection). CC_50_ HeLa = 153 ± 19 μM; CC_50_ BGM = 171 ± 16 μM; CC_50_ RD = 221 ± 28 µM

**Fig. 2:**
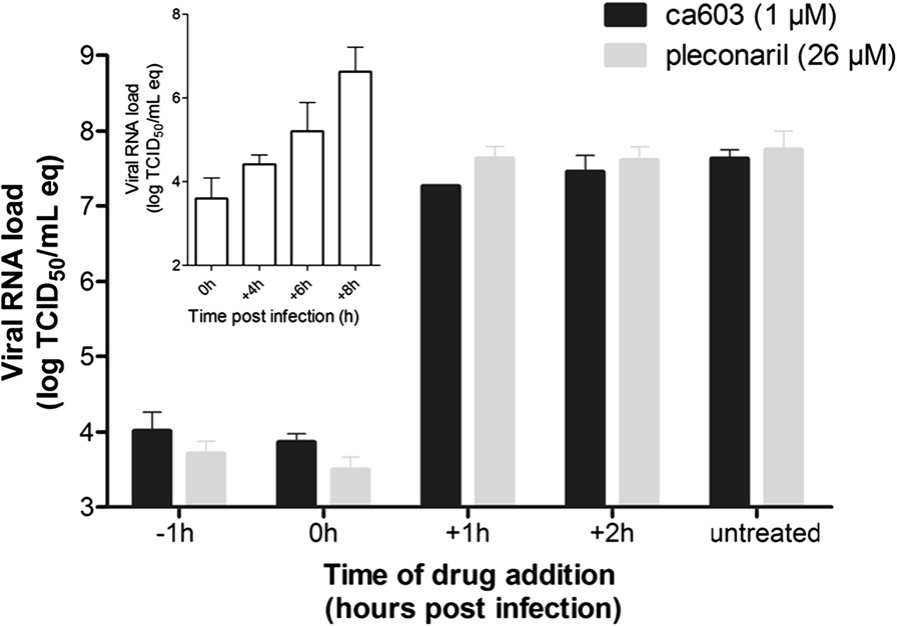
Compound ca603 (black) inhibits HRV14 replication only when added at an early stage and behaves similar to pleconaril (grey). Compounds were added prior to, at the time of, or after viral infection at indicated time points and viral RNA was quantified 8 h pi. Intracellular viral RNA load was expressed as equivalents of 50 % tissue infective dose/mL (log (TCID_50_/mL) eq.) (mean ± STD from two independent assays). Insert: Intracellular viral HRV14 RNA increased from baseline input (0 h) till 8 h post-infection (one full virus replication cycle)

Early-stage inhibitors like pleconaril and pirodavir are only able to prevent virus replication when added prior to, or at the time of infection in a time-of-drug addition assay. Akin to pleconaril, ca603 lost its inhibitory activity when added after infection, indicating that ca603 acts, as expected, at an early stage in the rhinovirus replication cycle (Fig. 2).

Next, the antiviral potency of ca603 was assessed on the replication of two earlier-reported pleconaril-resistant HRV14 variants [9]. Both virus mutants were generated by site-directed mutagenesis (Quikchange-Stratagene, primer sequences available on request) in a HRV14 infectious clone (kindly provided by D. Blaas, University of Vienna, Austria). Viruses derived from this infectious clone (IC) are designated as HRV14_IC_. The single mutant HRV14_IC_ VP1_A150V proved to be 10-and 40-fold less sensitive to ca603 and pleconaril, respectively (Table 2). Both ca603 and pleconaril proved also markely less active (12-and 28-fold, respectively) against the double mutant HRV14_IC_VP1_A150V_E276K.

**Table 2:** Reverse-engineered HRV14 virus mutants that carry a mutation in VP1 are less susceptible to the antiviral effect of ca603 and pleconaril. Sensitivity to the protease-inhibitor rupintrivir remained unchanged

HRV14_IC_	ca603		Pleconaril		Rupintrivir	
	EC_50_ (μM)	RR	EC_50_ (μM)	RR	EC_50_ (μM)	RR
Wild-type	0.014 ± 0.001	/	0.057 ± 0.004	/	0.0058 ± 0.0002	/
VP1_A150V	0.15 ± .0.02*	10	2.3 ± 0.1*	40	0.010 ± 0.001*	2
VP1_A150V_E276K	0.16 ± 0.02*	12	1.6 ± 0.2**	28	0.005 ± 0.0002	1

Activity was determined in a CPE reduction assay with MTS read-out. EC_50_ = median 50 % effective concentration ± MAD. Data are in duplicate from three independent assays. RR = relative resistance (EC_50_ of mutated strain / EC_50_ of wild-type). *p < 0.0001, **p < 0.001

**Fig. 3:**
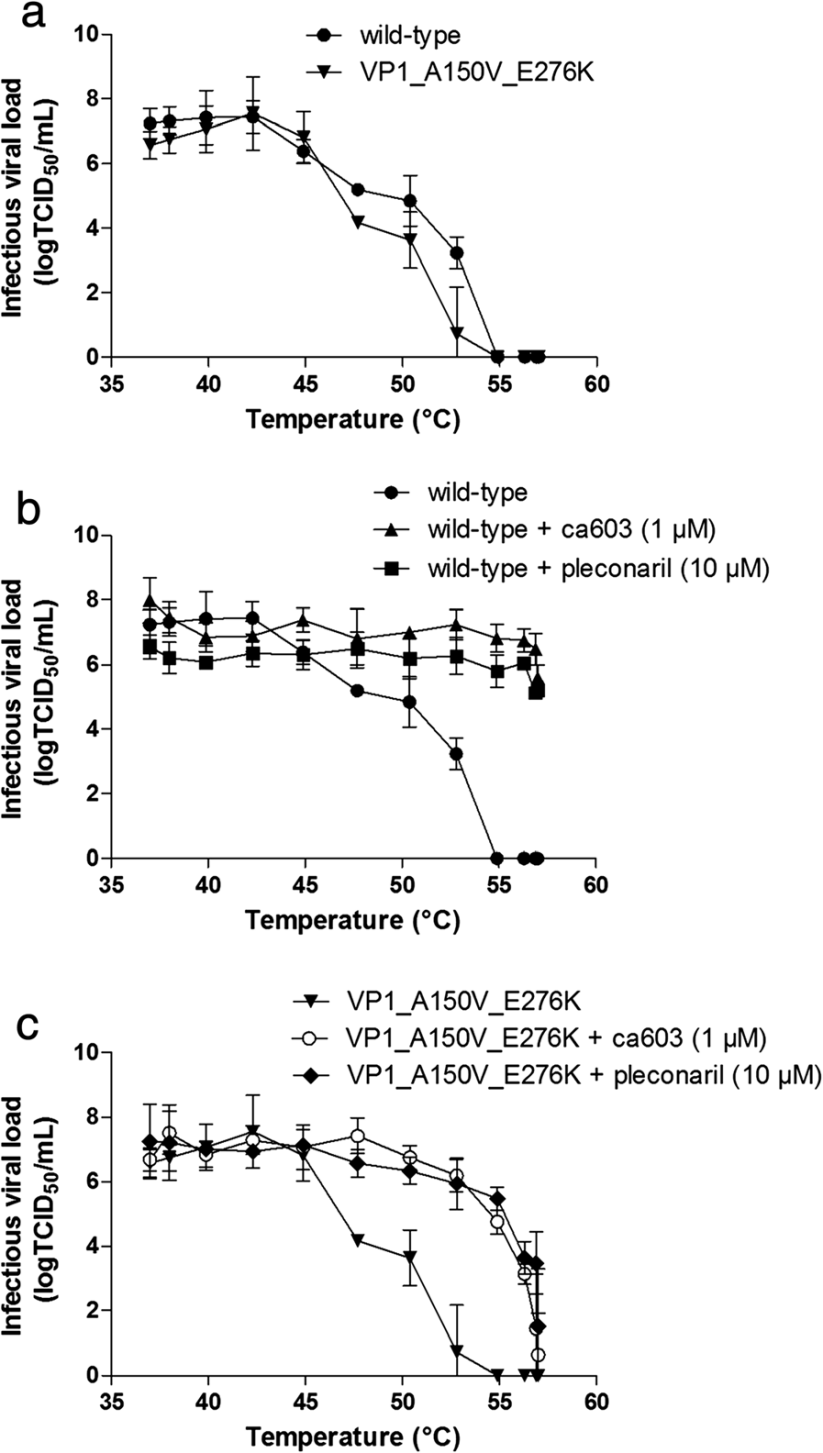
Effect of ca603 and pleconaril on heat-inactivation of HRV14. **a** HRV14_IC_wild-type and HRV14_IC_ VP1_A150V_E276K are equally inactivated by increasing temperatures. **b** Both ca603 and pleconaril were able to rescue wild-type virus from heat-inactivation. **c** The mutant VP1_A15V_E276K shows reduced protection from heat-inactivation in the presence of ca603 and pleconaril. Data are in duplicate from two independent assays (mean ± STD)

Rhinoviruses are sensitive to thermal stress. However, interaction with a capsid binder can stabilize the virion and increase the temperature at which heat inactivation occurs. We assessed the effect of ca603 on two HRV14_IC_ strains in a thermostability assay (Fig. 3). HRV14_IC_ wild-type or HRV14_IC_ VP1_A150V_E276K were incubated with a fixed concentration of ca603 (1 μM) or pleconaril (10 μM) for 15 min at 37 °C, 2 min at temperatures ranging from 37-57 °C (Lightcycler 96, Roche), followed by a rapid cool down to 4 °C. Infectious viral loads were subsequently quantified by end-point titration. Both HRV14_IC_ wild-type and HRV14_IC_ VP1_A150V_E276K were equally sensitive to heat-inactivation (Fig. 3a): gradual loss of infectivity is observed by increasing the temperature to 57 °C. In the presence of ca603 and pleconaril, heat-inactivation of HRV14_IC_ wild-type shifted to higher temperatures, suggesting that both compounds stabilize the viral capsid and protect the virus from heat degradation (Fig. 3b). Both compounds were not able to preserve the infectivity of HRV14_IC_ VP1_A150V_E276K to the same extent, indicating loss of interaction with either compound (Fig. 3c). Some residual protection was still observed, which is in agreement with the fact that the reverse-engineered pleconaril-resistant HRV strain is only partially resistant to the antiviral activity of the compounds.

**Fig. 4:**
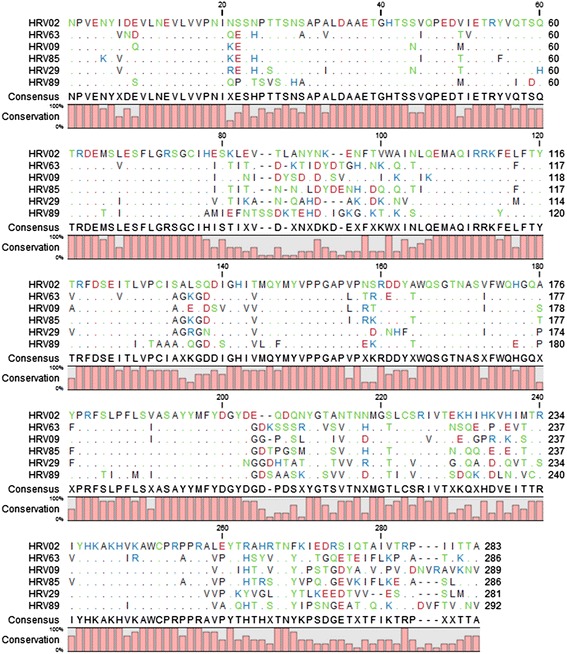
Sequence alignment of the HRV-A VP1 residues. Alignment was constructed with CLC sequence viewer (Qiagen)

Sequence alignment of the VP1 residues reported to constitute the hydrophobic pocket within the capsid were constructed with the use of CLC sequence viewer (Qiagen). For the HRV-B strains in the test panel, no satisfying explanation could be derived from this alignment that explains the moderate activity (>2 μM) against HRV42 and HRV70 compared to the potent activity (<2 μM) against HRV14 and HRV86 (Additional file 1: Figure S1). Also for the panel of HRV-A strains, no pattern of residues in the hydrophobic pocket could explain the difference in activity (from none against HRV2 and HRV63, moderate against HRV9 and HRV 85, to potent activity against HRV29 and HRV 89) (Additional file 2: Figure S2). The full alignment of HRV-A VP1 proteins revealed an alanine on position 176 (HRV02, FG loop) for the naturally compound-resistant strains, while a serine or threonine was detected for the strains against which moderate activity was observed, and a proline in the most susceptible strains (Fig. 4).

**Fig. 5:**
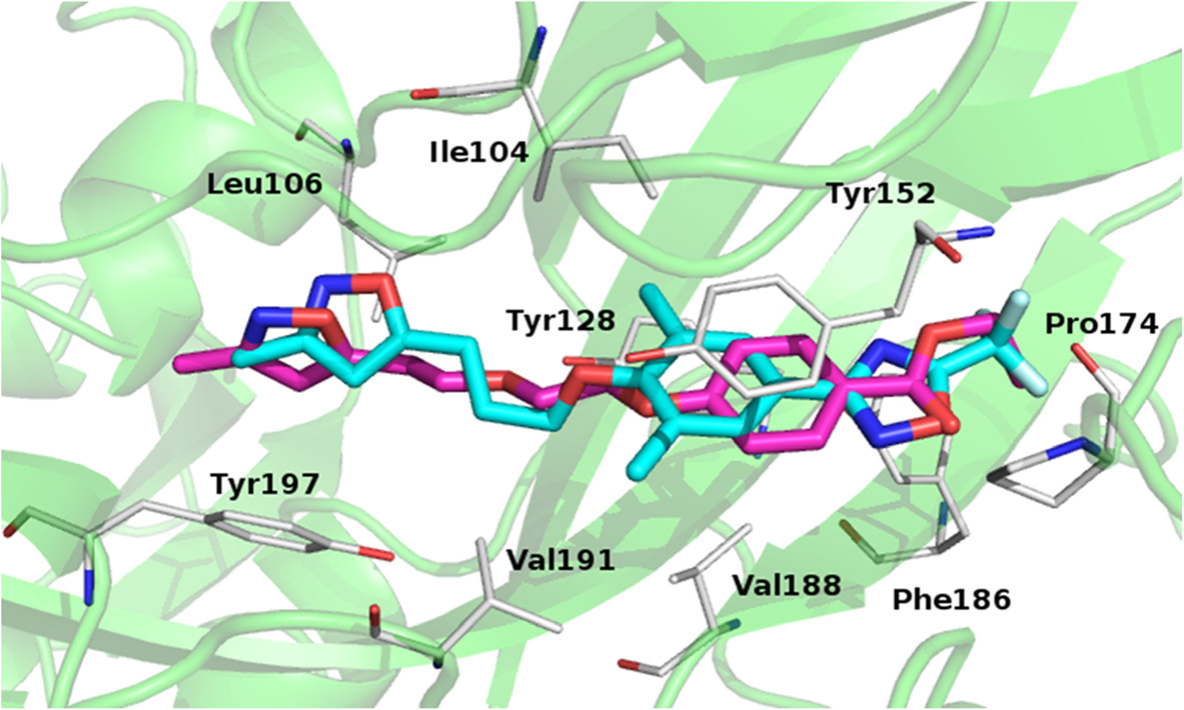
Plants-proposed binding mode for pleconaril (cyan) and ca603 (magenta) in the HRV14 VP1. Residues involved in direct interactions are depicted as white sticks

The cross-resistance and thermostabilizing studies indicate that the antiviral effect of ca603 is due to a pleconaril-like mechanism of action, i.e. acting as a capsid binder. The pleconaril/HRV14 capsid protein cocrystal structure (PDB id 1NCQ) [13] was used to study the binding mode of ca603. The Plants-proposed [14] ca603 docking pose well resembled the binding mode that was reported for pleconaril with the two derivatives sharing many key interactions. In particular for ca603, we observed that 1) the oxazole ring was stabilized by hydrophobic interactions with Y197 and L106; 2) the alkyl chain formed extensive van der Waals contacts with I104, V188 and V191; 3) the aryl ring established π-interactions with Y152 and Y128; and 4) the ethoxycarbonyl function made hydrophobic interactions mainly with F186 and P174 (Fig. 5). For a better understanding of how the observed mutations induce resistance, the interaction of the compound was also investigated in the context of a model in which the VP1_A150V mutant was introduced. Replacing the alanine at the bottom tip of the cleft with the bulkier valine narrowed the binding pocket, which causes a slight shift in binding mode. It should be noted that this shift did not disrupt the key binding interactions that were observed for wild-type HRV14 (Additional file 3: Figure S3). The modelling results therefore corroborate the biological results (i.e. a decrease in sensitivity but no full resistance) and validate the observed correlation between primary amino acid sequence of the binding site and the observed anti-HRV activity against wild-type and compound-resistant HRV14 variants.

Additional docking studies were carried out with the VP1 of HRV42, a virus serotype featuring the Y152F and V191L mutations that have been associated with resistance to pleconaril [15]. It was reported that the replacement of valine with the bulkier leucine narrowed the binding cleft with consequent negative steric effects [15]. Among the studied compounds, the pleconaril binding mode was markedly affected by the bulkier dimethyphenyl group close to position 191 (RMSD >2). On the contrary, the binding mode of ca603 seemed to be only weakly affected due to the smaller size of the unsubstituted phenyl ring near position 191, while all other interactions were retained, which is reflected in the anti-HRV42 activity of ca603 (Additional file 4: Figure S4).

## Additional files

Additional file 1: Figure S1. Sequence alignment of the HRV-B VP1 residues ligning the hydrophobic pocket (constructed with CLC sequence viewer (Qiagen)). Additional file 2: Figure S2. Sequence alignments of the HRV-A VP1 residues ligning the hydrophobic pocket (constructed with CLC sequence viewer (Qiagen)). Additional file 3: Figure S3. Top: Plants-proposed binding mode of ca603 in the HRV14 VP1_A150V (orange) and HRV14 wild-type (magenta) binding site. Bottom: Plants-proposed binding mode of pleconaril in the HRV14 VP1_A150V (orange) and HRV14 wild-type (cyan) binding site. Residues involved in direct interactions are depicted as white sticks, mutated residues are reported as pink sticks. Additional file 4: Figure S4. Plants-proposed binding mode of ca603 (yellow) in the HRV-B42 binding site; ca603 binding mode (magenta) in the HRV-B14 binding site is also shown. Residues involved in direct interactions are depicted as white sticks, mutated residues are reported as pink sticks.
