# Supplementary figures and images for: In vitro characterisation of a pleconaril/pirodavir-like compound with potent activity against rhinoviruses
Source: Virol J. 2015 Jul 14;12:106. doi: 10.1186/s12985-015-0330-4 (PMC4501209; doi:10.1186/s12985-015-0330-4)

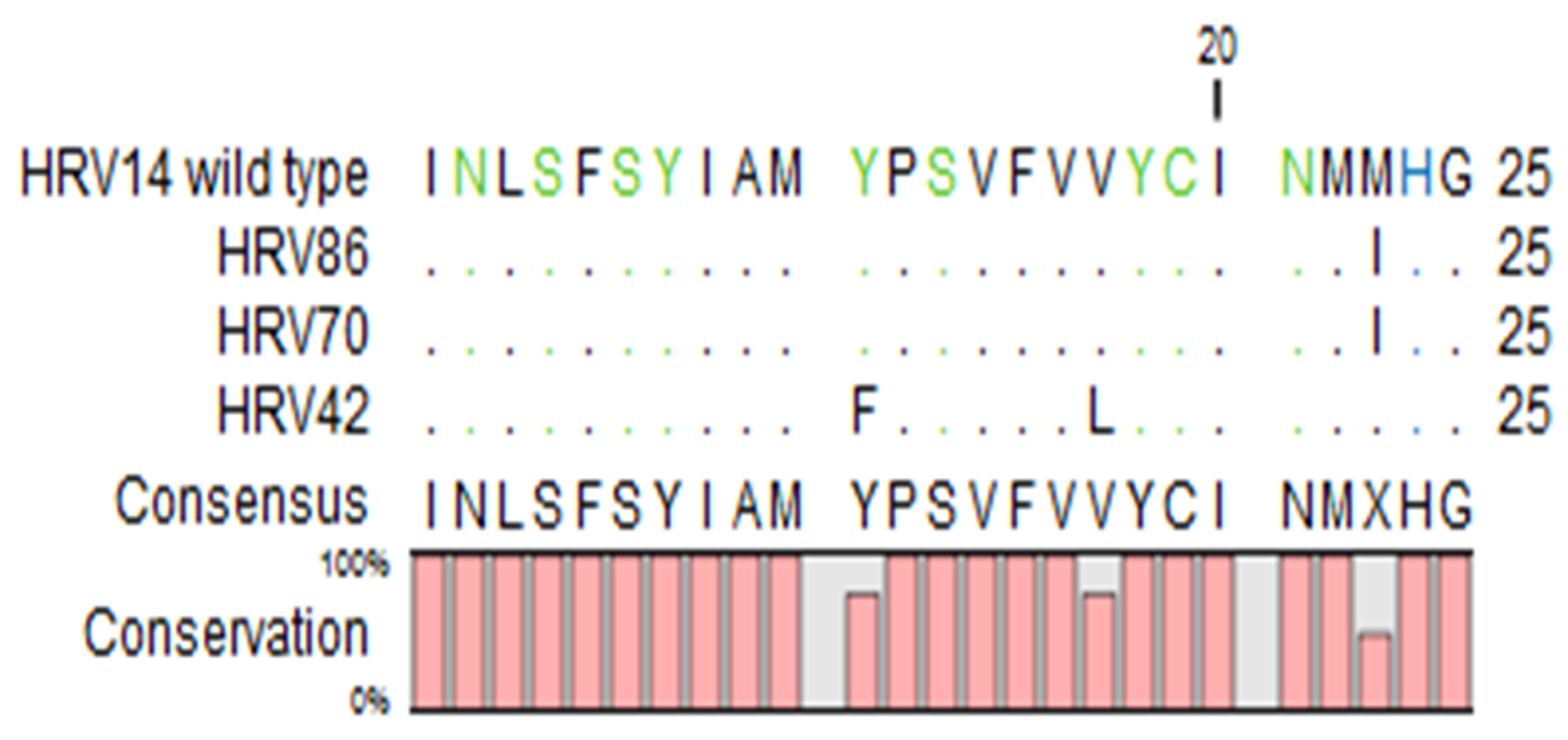

Supplement: Additional file 1: Figure S1. — Sequence alignment of the HRV-B VP1 residues ligning the hydrophobic pocket (constructed with CLC sequence viewer (Qiagen)). [file 12985_2015_330_MOESM1_ESM.tif]

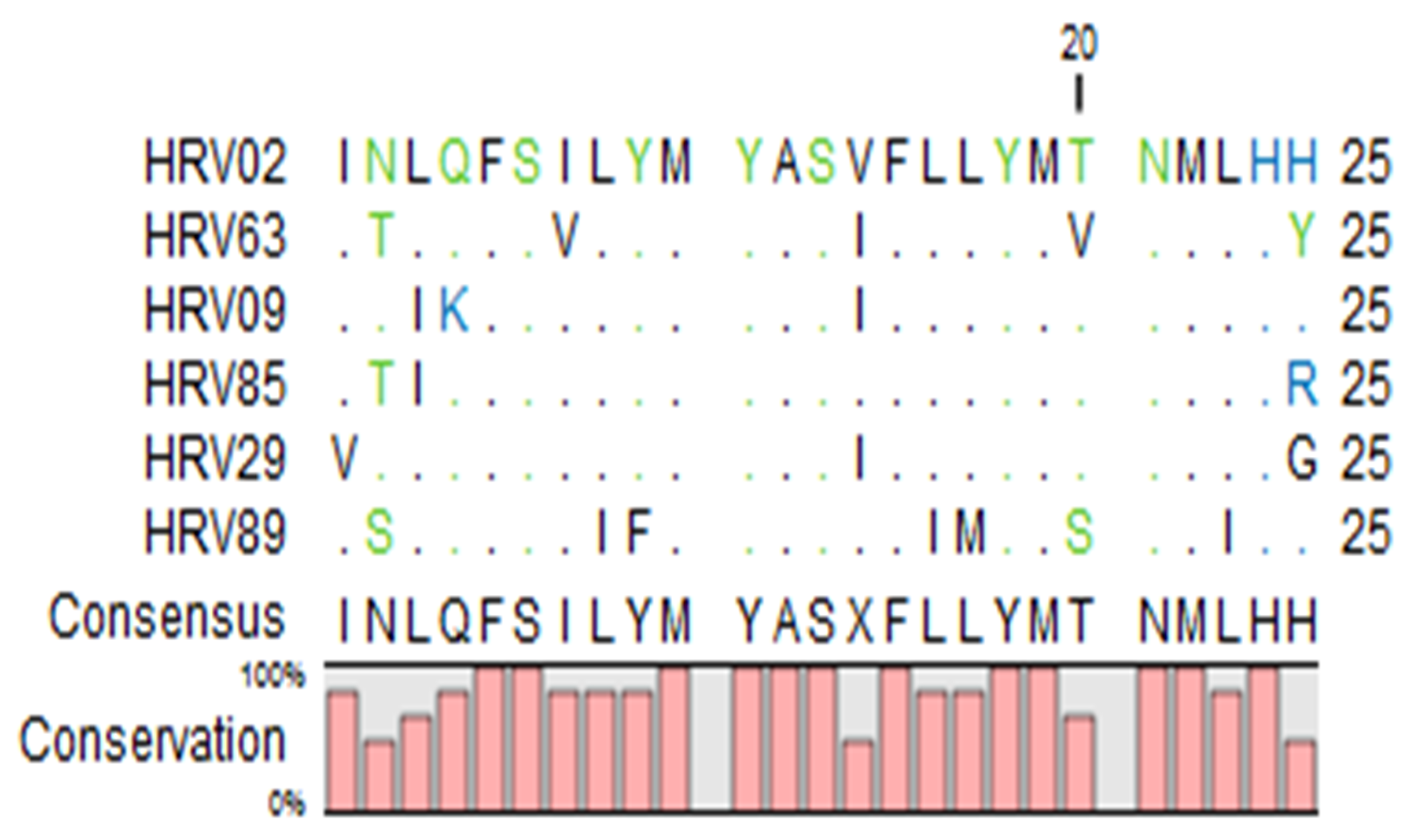

Supplement: Additional file 2: Figure S2. — Sequence alignments of the HRV-A VP1 residues ligning the hydrophobic pocket (constructed with CLC sequence viewer (Qiagen)). [file 12985_2015_330_MOESM2_ESM.tif]

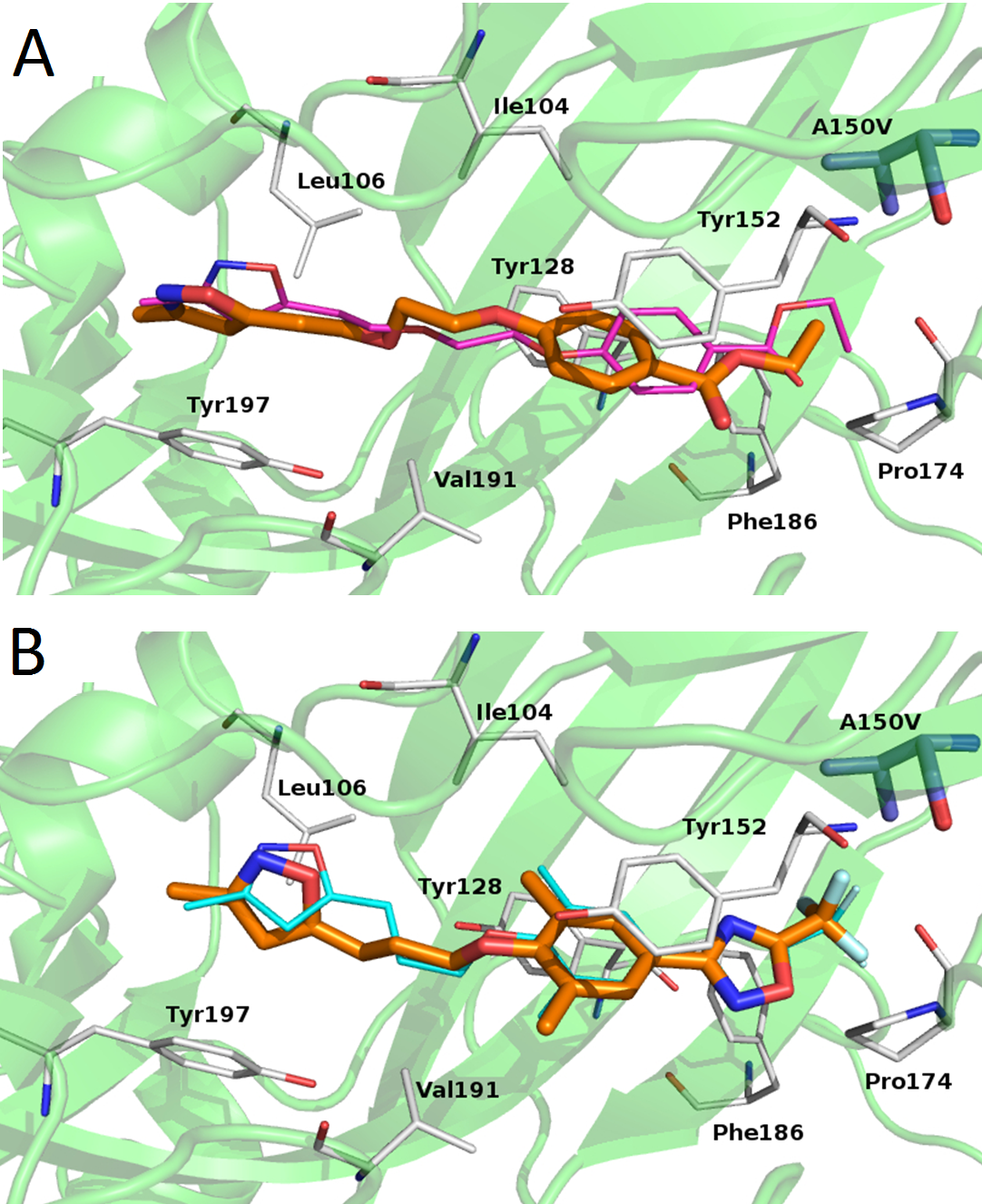

Supplement: Additional file 3: Figure S3. — Top: Plants-proposed binding mode of ca603 in the HRV14 VP1_A150V (orange) and HRV14 wild-type (magenta) binding site. Bottom: Plants-proposed binding mode of pleconaril in the HRV14 VP1_A150V (orange) and HRV14 wild-type (cyan) binding site. Residues involved in direct interactions are depicted as white sticks, mutated residues are reported as pink sticks. [file 12985_2015_330_MOESM3_ESM.tif]

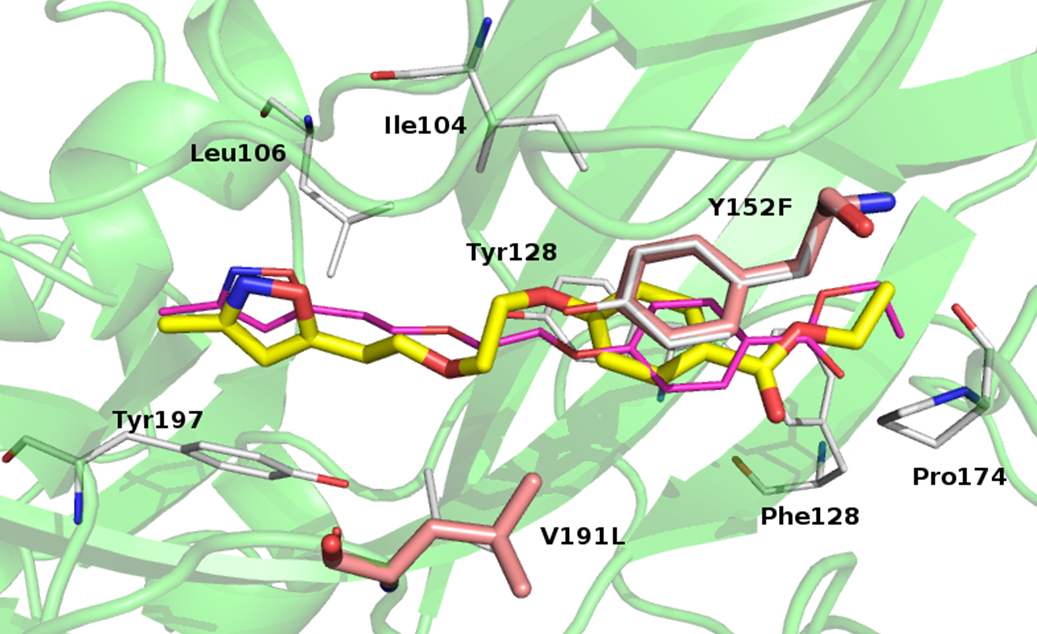

Supplement: Additional file 4: Figure S4. — Plants-proposed binding mode of ca603 (yellow) in the HRV-B42 binding site; ca603 binding mode (magenta) in the HRV-B14 binding site is also shown. Residues involved in direct interactions are depicted as white sticks, mutated residues are reported as pink sticks. [file 12985_2015_330_MOESM4_ESM.tif]
